# Effects of *Aggregatibacter actinomycetemcomitans* leukotoxin on endothelial cells^☆^

**DOI:** 10.1016/j.micpath.2013.05.001

**Published:** 2013-08

**Authors:** Anelia Dietmann, Alban Millonig, Valery Combes, Pierre-Olivier Couraud, Scott C. Kachlany, Georges E. Grau

**Affiliations:** a Department of Pathology, Vascular Immunology Unit, Sydney Medical School, The University of Sydney, 92-94 Parramatta Rd, Camperdown, 2050 NSW, Australia; b Department of Neurology, Innsbruck Medical University, Anichstrasse 35, 6020 Innsbruck, Austria; c Inserm, U1016, Institut Cochin, 22 rue Méchain, 75014 Paris, France; d Cnrs, UMR8104, Paris, France; e Univ Paris Descartes, Paris, France; f Department of Oral Biology, New Jersey Dental School, University of Medicine and Dentistry of New Jersey, 110 Bergen Street, P.O. Box 1709, Newark, NJ 07101-1709, USA

**Keywords:** Endothelium, Leukotoxin, *Aggregatibacter actinomycetemcomitans*, Apoptosis, Activation

## Abstract

*Aggregatibacter actinomycetemcomitans* is a human pathogen that produces leukotoxin (LtxA) as a major virulence factor. In this study the effect of LtxA on microvascular endothelial cell viability and phenotype was studied. High doses of single LtxA treatment (500 ng/ml to 5 μg/ml) significantly and irreversibly decreased cell proliferation and induced apoptosis, as assessed by tetrazolium salt and annexin V assay, respectively. Apoptosis was partially inhibited by the pan-caspase inhibitor, z-VAD-fmk. LtxA caused a cell cycle arrest in the G2/M phase after 72 h. Between 500 ng/ml and 5 μg/ml, after long- or short-term stimulation LtxA increased the expression of ICAM-1 and VCAM-1, as well as the percentages of endothelial cells expressing these adhesion molecules. Thus, *A. actinomycetemcomitans* LtxA has substantial pro-inflammatory effects on human brain endothelial cells by upregulation of ICAM-1 and VCAM-1. Furthermore, LtxA in higher concentration was found to decrease proliferation and induces apoptosis in microvascular endothelial cells.

## Highlights

• LtxA has anti-proliferative effects on endothelial cells.
• LtxA induces a G2/M phase cell cycle arrest in endothelial cells.
• LtxA induces apoptosis in endothelial cells.
• LtxA increased expression of ICAM-1 and VCAM-1 in endothelial cells.

## 1 Introduction

*Aggregatibacter actinomycetemcomitans* is an inhabitant of the oral cavity and periodontal pathogen [1], [2]. In periodontal disease, the bacterium infects and proliferates within the periodontal pocket, between the gingival tissue and the tooth. The presence of bacteria and their products such as secreted proteins and LPS induce an inflammatory response by the host. Inflammation leads to tissue damage and alveolar bone loss that is characteristic of periodontal diseases [3]. *A. actinomycetemcomitans* has been highly associated with a rapidly progressing form of periodontal disease known as localized aggressive periodontitis (LAP) that occurs in adolescents [1], [4]. This bacterium has also been reported to cause non-oral infections such as pneumonia, endocarditis, pericarditis, bacteremia, septicemia, osteomyelitis, synovitis, infectious arthritis, skin infections, urinary tract infections and brain abscesses [4], [5], [6].

A major virulence factor of *A. actinomycetemcomitans* is the secretion of leukotoxin (LtxA), which induces apoptosis in white blood cells (WBC) from humans and Old World primates [7], [8], [9], [10]. Apoptosis induction by LtxA occurs via different pathways such as a mitochondrial signaling pathway that results in collapse of the mitochondrial membrane potential and arrest of oxidative phosphorylation [11], [12], [13] or by activation of caspase 1 [14]. Furthermore, LtxA has been shown to induce G2/M cell cycle arrest and apoptosis in mouse B-cell hybridoma HS-72 cells [15]. However, the molecular pathway that leads to LtxA induced cellular apoptosis and cell cycle arrest is not well understood. LtxA is believed to play a crucial role in evasion of the host immune response by the bacterium. LtxA likely exerts its effects within the periodontal pocket where polymorphonuclear leukocytes and other immune cells infiltrate to control the infection. The receptor for LtxA on WBCs is leukocyte function antigen-1 (LFA-1; CD11a/CD18) [16], [17], [18]. LFA-1 is expressed only on WBCs and is normally involved in migration of WBCs to infected and injured tissues [19], [20]. When presented in its activated or “exposed” state, LFA-1 binds intercellular adhesion molecule-1 (ICAM-1) on the surface of vascular endothelial cells resulting in adhesion of WBCs to the endothelial lining and subsequent extravasation. Recently, we reported that LtxA preferentially targets immune cells expressing the activated form of LFA-1, resulting in selective depletion of host cells [7].

While studying the interaction between WBCs and vascular endothelial cells, we found that relatively high doses of LtxA irreversibly damaged endothelial cells and caused changes in expression levels of endothelial adhesion molecules. This work provides a novel mechanism for *A. actinomycetemcomitans*-induced tissue damage during infection.

## 2 Material and methods

### 2.1 Purification of LtxA

Leukotoxin (LtxA) was purified from culture supernatants of *A. actinomycetemcomitans* strain NJ4500 as previously described [7], [21]. The storage buffer for the purified toxin was 20 mM Tris–HCl, pH 6.8, 250 mM NaCl, and 0.2 mM CaCl2. The typical yield was 0.5 mg/100 ml starting culture. For long-term storage (greater than one month), protein was lyophilized in sterile glass vials and stored at −80 °C. Samples were reconstituted in sterile distilled water prior to use and we found that when stored in this manner, LtxA was stable for at least 6 months. All toxin preparations were filtered through a 0.22 μm filter prior to use.

For experimental setup heat inactivation (65 °C for 20 min) has been shown effectively abolishing all toxic effects of LtxA. Detailed description has been published elsewhere [7].

### 2.2 Cell culture

In this study, human microvascular endothelial cells (immortalized cell line hCMEC/D3 were used [22] at a passage number 28–32. hCMEC/D3 were grown in EBM-2 medium (Lonza CC-3156), supplemented with 5% fetal bovine serum, 1.4 μM hydrocortisone, 5 μg/ml ascorbic acid, 1% chemically defined lipid concentrate, 10 mM HEPES, and 1 ng/ml human basic fibroblast growth factor.

### 2.3 Proliferation assays and cell count

After trypsinization, cells were seeded in 96-well plates pre-coated with 0.3% collagen (5000 cells/well). Medium was supplemented with LtxA at concentrations of 5 μg/ml, 500 ng/ml or 50 ng/ml or corresponding to the highest dosage LtxA-Buffer alone was added. After 24, 48, 72, 96 and 144 h, proliferation was quantitated using the CCK-8 Assay, based on the mitochondrial reduction of tetrazolium salt (Fluka 96992). Briefly, medium was removed and 100 μl of fresh medium and 10 μl of CCK-8 solution was added. Absorbance was measured after 4 h of incubation with a BMG FluoStar OPTIMA spectrofluorophotometer.

Endothelial proliferation was also assessed by enumeration. To this end, between 50,000 and 60,000 cells were seeded per well in a collagen coated 12-well plate without treatment or with immediate addition of LtxA-Buffer, or LtxA at 5 μg/ml, 500 ng/ml, 50 ng/ml or 5 ng/ml. Seventy-two or 96 h after seeding with or without treatment, cells were trypsinized, washed and counted in a Malassez haematocytometer in triplicates and results were expressed as cells/cm^2^. All assays were repeated 3–5 times.

### 2.4 Cell cycle and apoptosis

hCMEC/D3 cells were grown in pre-coated 12-well plates without treatment, with LtxA-Buffer or with LtxA at doses ranging from 5 μg/ml to 5 ng/ml for 24, 72 or 96 h. Cells were harvested by trypsinization, washed in RPMI containing 10% FCS, then washed in ice-cold PBS and resuspended in 1 ml 80% ethanol. Cells were fixed at −20 °C overnight. Ethanol was removed after centrifugation and cells were stained in PBS, containing 0.05% Triton-X, 0.1 mg/ml RNAse A and 15 μl Propidium Iodide for 1 h on ice. After this, cells were resuspended in 3 ml PBS, pelleted by centrifugation and resuspended in 500 μl PBS for flow cytometric analysis, performed in duplicates (except 24 h experiment), repeated 3–4 times.

For analysis of apoptosis, cells were grown in 6-well plates without changing of medium for 48 or 72 h without treatment, with LtxA-Buffer or with LtxA at doses ranging from 5 μg/ml to 5 ng/ml. The pan-caspase inhibitor, z-VAD-fmk, at a final concentration of 25 μM was added to high concentrations of LtxA (5 μg/ml and 500 μg/ml) to evaluate apoptosis via caspase activation. Supernatants and cells were collected, pelleted and washed in PBS twice. Cells were then stained in binding buffer for annexin V-FITC (Beckman Coulter ApoScreen) and 7-AAD (BD) for 15 min at room temperature. Another 100 μl of binding buffer was added and cells were directly analyzed by flow cytometry (Beckman Coulter FC-500). All assays were performed in triplicates and repeated 3 times.

For fluorescence microscopy, cells were grown on tissue culture dishes with cover glass bottom (Fluorodish FD 35-100). Cells were either untreated or treated with 5 μg/ml LtxA. After 72 h cells were washed with PBS and stained with Hoechst 33258 (0.01 mg/ml) for 20 min. Cells were washed again and images were taken directly afterwards at 40× magnification (Olympus IX-71).

### 2.5 Apoptotic microparticles

hCMEC/D3 cells were grown in 6-well plates for 48 h without changing of medium and in the presence or not of LtxA 5 ug/ml to 5 ng/ml or LtxA-Buffer. After 24 h and 48 h 90 μl of supernatant was removed and stained for annexin V (ApoScreen Beckman Coulter) according to Combes et al. [23]. Analysis of Annexin V positive MP was performed in triplicates, repeated 3 times.

### 2.6 Analysis of cell activation

For analysis of long-term effects of LtxA, hCMEC/D3 cells were seeded in collagen-coated 24-well or 12-well plates with or without LtxA at 5 μg/ml, 500 ng/ml, 50 ng/ml, 5 ng/ml or LtxA-Buffer. Medium was changed every other day and cells were grown until untreated wells were confluent (three to four days). For short-term LtxA effect evaluation, hCMEC/D3 were grown in collagen-coated 24-well or 12-well plates until confluence and then treated for 16 h with LtxA at 5 μg/ml, 500 ng/ml, 50 ng/ml, 5 ng/ml, LtxA-Buffer or medium alone. Cells were stained for CD54 (mAb from Beckman Coulter IM1239U) and CD106 (mAb from eBioscience 12-1069-73) and analyzed by flow cytometry in duplicates, repeated 3–4 times.

### 2.7 Statistics

All results are presented as mean values and SEM unless otherwise indicated. Treatment groups were compared by ANOVA and Bonferroni post test between groups. Comparison between treatment groups at different time points were performed by two-way ANOVA and Bonferroni post test between groups. Values *p* < .05 were considered significant. Graphs and descriptive statistics were performed using GraphPad Prism 5.

## 3 Results

### 3.1 Purification and activity of LtxA

**Fig. 1:**
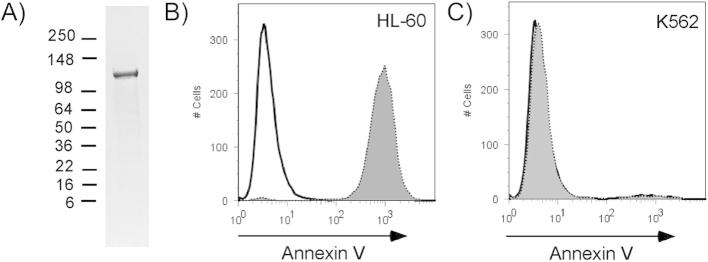
Purification and activity of LtxA. LtxA was purified from culture supernatants as described in Materials and Methods. (A) SDS-PAGE gel of purified LtxA. The gel was stained with GelCode blue reagent. Purified LtxA was incubated with HL-60 cells (B) or K562 cells (C), which lack LFA-1 expression. Cells were stained with Annexin V and analyzed by flow cytometry. The solid line represents buffer-treated cells and the dashed line/shaded curve represents after treatment with LtxA for 24 h.

LtxA was purified from culture supernatants of *A. actinomycetemcomitans* (Fig. 1A). To confirm that our preparation contained only LtxA and not other products that could potentially affect cells (eg. LPS, cytolethal distending toxin, Endotoxin), LtxA (5 μg/ml) was incubated with HL-60 cells and K562 cells. K562 cells are a white blood cell line that does not express LFA-1 and are therefore resistant to LtxA-mediated cytotoxicity. Apoptosis was assessed via annexin V staining and flow cytometry (Fig. 1B and C). Nearly all the HL-60 cells were annexin V positive, indicating they were undergoing apoptosis (Fig. 1B). In contrast, K562 cells did not stain with annexin V after LtxA treatment and the buffer- and LtxA-treated curves were superimposable. Thus, cytotoxicity was due to LtxA in our purified preparation.

### 3.2 LtxA reduces endothelial cell proliferation

**Fig. 2:**
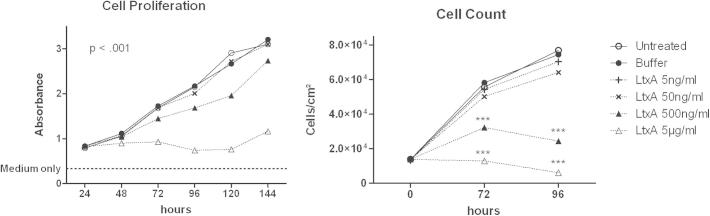
Effect of LtxA on cell proliferation. hCMEC/D3 cells were treated with purified LtxA, buffer, or were untreated. Cell proliferation was analyzed with a tetrazolium based assay every day after seeding or by cell count after 72 and 96 h. Mean values are shown for every time point. Statistical testing was performed by two-way ANOVA with Bonferroni post test. ***p < .001 compared to untreated cells.

To assess the effect exerted by purified LtxA (Fig. 1) on human brain endothelial cells (hCMEC/D3) proliferation, cells were treated once with increasing concentrations of LtxA (5 ng/ml–5 μg/ml) and grown for up to six days. Every day a tetrazolium-salt-based assay (CCK-8) was performed as well as cell counts. Proliferation was irreversibly abrogated by a single treatment of high dose LtxA (5 μg/ml). At 500 ng/ml a significant decrease in proliferation was observed whereas lower LtxA concentrations or LtxA-Buffer had no effect (Fig. 2). Whereas untreated cells as well as LtxA-Buffer and low dosage LtxA treated cells quintupled after 96 h, 5 μg/ml LtxA reduced cell numbers by half, and 500 ng/ml LtxA resulted only in a duplication of cell numbers at 96 h. As shown in Fig. 6, hCMEC/D3 cells presented with dramatic morphological changes when treated with a single dose of 5 μg/ml LtxA. Cells increased substantially in size and developed polyploidy. Monolayer formation and even generation of cell–cell contacts seemed to be inhibited by LtxA treatment and could not be observed.

### 3.3 LtxA induces cell cycle arrest in G2/M phase

**Fig. 3:**
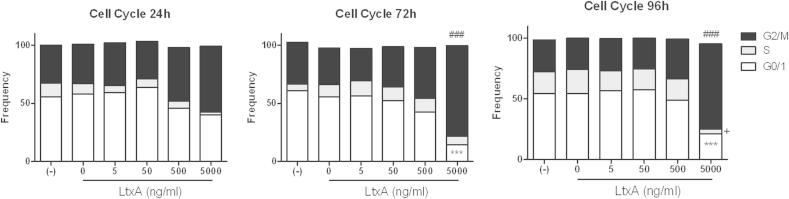
LtxA effect on cell cycle. hCMEC/D3 cells were treated with LtxA, buffer, or were untreated and cultured for 24, 72, or 96 h. Proportions of cell cycle phases were measured by propidium-iodide staining and flow cytometry. ANOVA and Bonferroni post tests were used for proportions of G0/1 (***p < .001), S (+*p* < .05), G2/M (###*p* < .001) compared to untreated cells.

hCMEC/D3 cells were treated once with increasing concentrations of LtxA (5 ng/ml–5 μg/ml) and cell cycle analysis was performed at different time points (24, 72, and 96 h). Treatment with LtxA dose-dependently increased the proportion of cells in the G2/M phase but decreased the proportion in the G1 phase (Fig. 3). After 96 h, 54% of untreated or LtxA-buffer treated cells were in the G1 phase and 26% in the G2/M phase, compared to 21% of cells in the G1 phase and 70% in the G2/M phase when treated with 5 μg/ml LtxA (*p* < .001, *p* < .001, respectively). The S phase was reduced from 18% in untreated or LtxA-buffer treated cells to 3% in LtxA treated cells (*p* < .05).

### 3.4 LtxA induces apoptosis in hCMEC/D3 cells in a partially caspase-dependent way

**Fig. 4:**
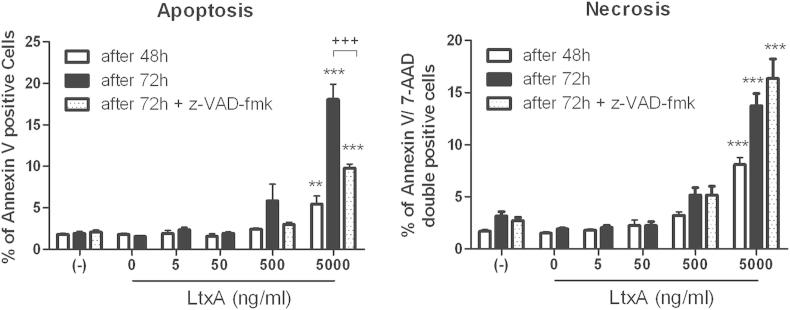
Apoptosis and necrosis induction by LtxA. hCMEC/D3 were cultured with LtxA or buffer or untreated for 48 or 72 h and apoptosis or necrosis was analyzed by Annexin-V and 7-AAD staining and flow cytometry. A pan-caspase inhibitor z-VAD-fmk was added for 72 h to untreated cells as well as cells treated with high doses of LtxA (500 ng/ml and 5 μg/ml). Data represents mean values and SEM. Significance was tested using ANOVA and Bonferroni post test (***p < .001, **p < .01 tested vs. untreated group; +++*p* < .001 tested within group vs. z-VAD-fmk).

Single dose LtxA treatment (5 μg/ml) significantly increased numbers of apoptotic cells (annexin V positive, 7-AAD negative) after 48 h and 72 h (Fig. 4). After 72 h a mean of 1.9% of hCMEC/D3 stained positively for annexin V and were therefore considered apoptotic, compared to a mean of 18% in LtxA 5 μg/ml treated cells (*p* < .001) and 5.9% in LtxA 500 ng/ml treated cells (NS). Addition of the pan-Caspase inhibitor z-VAD-fmk (25 μM) to the 5 μg/ml LtxA treatment for 72 h reduced apoptotic cell proportions to 9.8% (*p* < .001). Experiments with z-VAD-fmk were only performed for 5 μg/ml and 500 ng/ml LtxA treatment or untreated cells for 72 h. After 48 h and 72 h, LtxA treatment (≥500 ng/ml) significantly increased numbers of annexin V and 7-AAD double positive cells (after 72 h 13.7% in 5 μg/ml [*p* < .001] and 5.2% [NS] in 500 ng/ml) were found, compared to untreated (3.2%), LtxA-buffer treated (1.9%) or LtxA in lower concentrations (2.2% in 50 ng/ml, and 2% in 5 ng/ml, respectively). These cells were considered necrotic cells. Treatment with z-VAD-fmk concomitantly with to LtxA 5 μg/ml or 500 ng/ml did not reduce numbers of necrotic cells (16.4% and 5.2%, respectively, untreated 3.2%).

**Fig. 5:**
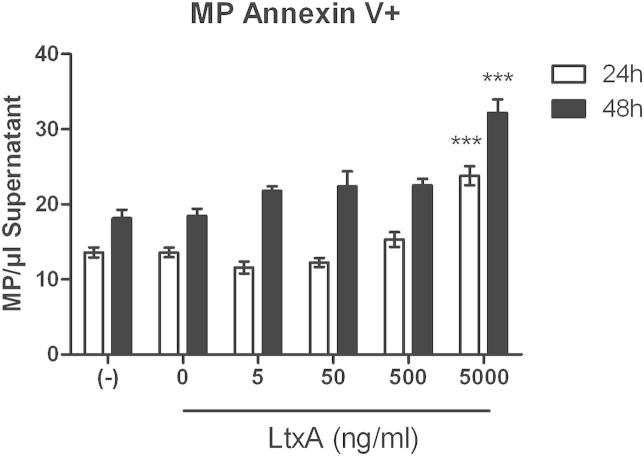
Apoptotic microparticles. Supernatants of hCMEC/D3 untreated or treated with increasing concentrations of LtxA or LtxA-Buffer for 24 h and 48 h were stained with Annexin V and endothelial microparticles were analyzed by flow cytometry. Means and SEM, ANOVA and Bonferroni post test, ***p < .001 (Figure shows significances only for testing against untreated cells).

Since cellular microparticles positively staining for annexin V are considered markers of early apoptosis we evaluated numbers of endothelial microparticles after 24 h and 48 h incubation with LtxA at increasing concentrations and LtxA-buffer. After 24 h and 48 h increased numbers of annexin V positive microparticles at 5 μg/ml LtxA treatment (both *p* < .001) were found, indicating early signs of apoptosis and/or cellular activation already after 24 h (Fig. 5).

**Fig. 6:**
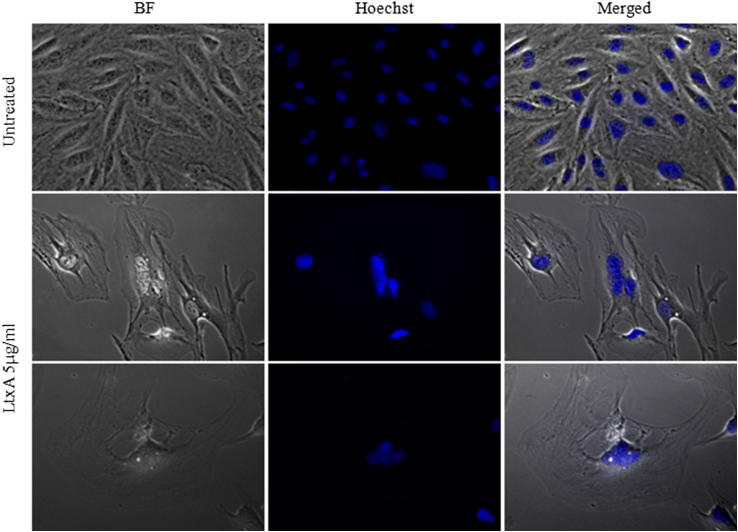
Morphological changes induced by LtxA. Representative morphology of hCMEC/D3 cells cultured with the LtxA. hCMEC/D3 cells were cultured untreated or in the presence of 5 μg/ml LtxA for 72 h and stained with the DNA-specific fluorochrome Hoechst dye 33258. Micrographs were captured at 40× magnification. BF, brightfield microscopy.

After 72 h apoptotic endothelial cells, exhibiting the characteristic chromatin condensation were observed by Hoechst staining and fluorescence microscopy (Fig. 6).

### 3.5 LtxA activates endothelial cells

**Fig. 7:**
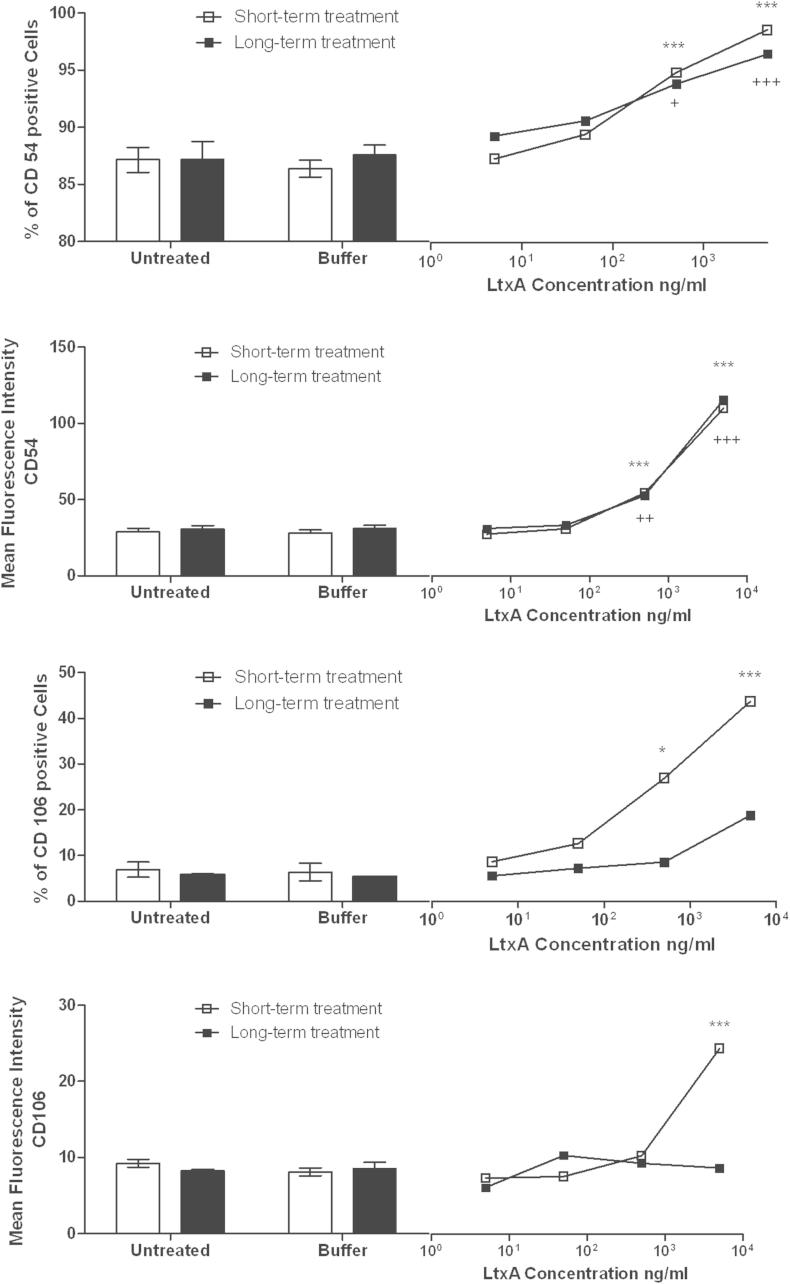
Activation of adhesion molecules. hCMEC/D3 cells were seeded in the presence of a single LtxA dose or buffer (long-term) or confluent hCMEC/D3 monolayers were treated with LtxA or buffer for 16 h (short-term). When untreated cells reached confluency (long-term), or after overnight stimulation (short-term), cells were harvested and stained for cellular adhesion molecules CD54 (ICAM-1) or CD106 (VCAM-1). Percentages of CD54 or CD106 positive cells as well as CD54 and CD106 mean fluorescence intensity are shown for untreated, buffer-treated, or LtxA-treated cells. Data represents means and SEM. ANOVA was used for each treatment group. * refers to the short-term treatment groups, + refers to the long-term treatment groups. Figure shows significances for testing against buffer-treated group only. */+*p* < .05, **/++*p* < .01, ***/+++*p* < .001.

As shown in Fig. 7, hCMEC/D3 cells either seeded with or without a single dose of LtxA (long-term) in increasing concentrations (5 ng/ml to 5 μg/ml) or treated with LtxA for 16 h after forming a confluent monolayer (short-term), expressed increased levels of both ICAM-1 (CD54) and VCAM-1 (CD106). Percentages of ICAM-1 positive cells as well as mean fluorescence intensity (MFI) were comparably and dose-dependently upregulated in both long- and short-term treatments. After short-term treatment of a confluent monolayer 87.2% of untreated cells compared to 95.3% of LtxA 500 ng/ml and 99% of LtxA 5 μg/ml treated cells expressed ICAM-1 (long-term ICAM-1 positive cells untreated 87.2%, LtxA 500 ng/ml 94.2% and LtxA 5 μg/ml 96.9%). Mean ICAM-1 MFI of untreated cells was 28.9 and 119.9 (long-term MFI untreated 30.7 vs. LtxA 5 μg/ml 126) after LtxA 5 μg/ml treatment. VCAM-1 expression was dose-dependently higher when hCMEC/D3 confluent monolayers were treated for a short-term period (16 h). After a 16 h LtxA treatment 6.9% (MFI 9.2) of untreated cells expressed CD106 compared to 26.9% (MFI 11.4) of LtxA 500 ng/ml and 43.7% (MFI 26.9) of LtxA 5 μg/ml treated cells. When cells were grown in the presence of LtxA 5.7% (MFI 8.2) of untreated cells and 8.6% (MFI 10.3) of LtxA 500 ng/ml or 18.8% (MFI 9.7) of LtxA 5 μg/ml treated cells expressed CD106 (Fig. 7).

## 4 Discussion

Many pathogenic bacteria produce toxins that amplify or suppress the host immune response by altering cell signaling or transcriptional responses. Evidence to date suggests that *A. actinomycetemcomitans* LtxA disrupts the host immune response mainly by killing of host immune cells [7]. Our present findings of LtxA-induced apoptosis and activation of microvascular endothelial cells, never previously reported, amplify the spectrum of pathogenetic mechanisms of *A. actinomycetemcomitans* and provide further explanation for tissue destruction in localized aggressive periodontitis and other diseases associated with this bacterium.

As the interface between circulation and site of infection, the vascular endothelium plays a pivotal role in recruitment of leucocytes and launch of immune responses, as well as in the basic function of blood circulation and tissue maintenance. We found that a single dose of purified LtxA administered to human microvascular endothelial cells (hCMEC/D3) importantly and irreversibly inhibits cell proliferation by G2/M cell cycle arrest. In addition LtxA induces apoptosis, which is partially caspase-dependent. Both decreased cell viability and apoptotic cell death of endothelial cells due to LtxA could lead to degeneration of microvasculature and subsequently to gingival tissue destruction. Impairment of the endothelial barrier function would facilitate tissue invasion and distribution of *A. actinomycetemcomitans* and LtxA [24], [25]. Indeed, Loesche [25] postulated that LtxA is the causative agent of local tissue destruction during LAP associated with *A. actinomycetemcomitans*. In addition, intact blood perfusion of tissue surrounding the sites of inflammation is crucial for an effective host immune response. Damage to the endothelial lining of microvessels by LtxA could severely compromise circulation and impair host defense to infection, further contributing to the pathogenic nature of the bacterium. However, in our study endothelial cells were only affected by LtxA in a relatively high dose rage from 500 ng/ml to 5 μg/ml, whereas LFA-1 bearing cells such as macrophages or human PBMCs were already affected at 1–10 ng/ml [7], [26] and minimal amounts of LtxA have been shown to induce a rapid proinflammatory reaction in human macrophages, already at a ratio of 1 bacterium/macrophage [27]. To our knowledge concentrations of LtxA locally present in patients with LAP is not known, however, currently it is not evaluable if higher dosage as used in our experiment do have important clinical relevance.

Already after 24 h of LtxA treatment in the highest concentration, hCMEC/D3 cells shed significantly increased amounts of annexin V positive microparticles into the cell culture supernatant. Cellular microparticles are regarded as reliable markers of cell stress and apoptosis [28], [29]. Therefore, our finding of increased endothelial microparticle numbers is a clear sign of cell activation and/or apoptosis as an immediate reaction to LtxA treatment.

It has been reported that apoptosis of endothelial cells *in vitro* is associated with the establishment of a pro-inflammatory milieu leading to paracrine induction of ICAM-1 and VCAM-1 and, in turn, increased adhesiveness resulting in adhesion and transmigration of monocytic cells into the vessel wall [30]. In our study, we found that levels of ICAM-1 and VCAM-1 increased on endothelial cells upon treatment with LtxA. Leukocyte recruitment form the circulation to sites of inflammation and infection involves a multistep cascade consisting of leukocyte rolling, firm adhesion, and, ultimately, transmigration [31]. A key step in this process is the interaction of ICAM-1 with its leukocyte counter receptor, lymphocyte function-associated antigen-1 (LFA-1) [32], [33], [34]. Whereas under physiological conditions vascular endothelium expresses low levels of ICAM-1, inflammatory stimuli can significantly increase ICAM-1 surface expression. In acute and chronic inflammatory diseases, endothelial cells become activated and express increased levels of ICAM-1, in addition to VCAM-1 and E-selectin [35], [36], [37]. VCAM-1 is implicated in the control of leukocyte rolling in the beginning of leukocyte recruitment process whereas ICAM-1 accounts for firm arrest [38]. This might be reflected by our results of VCAM-1 up-regulation in short-term stimulation whereas ICAM-1 was upregulated in short- and long-term stimulation.

Whether the upregulation of ICAM-1 and VCAM-1 upon LtxA treatment is directly induced by LtxA or caused by paracrine induction due to a pro-apoptotic milieu caused by the toxin has to be further investigated. However, other pro-inflammatory effects of LtxA such as the activation and secretion of interleukin-1 beta have already been described [27]. It is therefore tempting to speculate that an autocrine stimulation of endothelial cells by their own IL-1 could play a role in the observed CAM upregulation.

Epidemiologic and clinical studies suggest a connection between poor oral health and increased risk of cardiovascular disease (CVD) [39]. Perodontopathogens have been found in atherosclerotic plaques [40] and *A. actinomycetemcomitans* periodontitis has been linked to a higher risk of cardiovascular diseases and atherosclerosis [41]. Recently Zhang et al. described elevated ICAM-1 expression in the aorta of mice which have been systemically challenged with *A. actinomycetemcomitans* [42]. Endothelial ICAM-1 upregulation is of special interest since it is one of the basal mechanisms associated with atherosclerotic plaque formation and subsequentially development of CVD [43], [44].

Since endothelial cells do not express LFA-1, a β2 integrin receptor and the natural receptor for LtxA, it is not clear how LtxA may interact with this cell type. However endothelial cells do express β1 and β3 heterodimers on their cell surfaces [45]. While no significant amino acid homology between β1/β3 and β2 integrins is known, a certain amount of structural homology does exist [46]. Therefore, even though the toxin does not bind with a high affinity to β1 or β3 as it does to β2 high LtxA dosage might be enough binding stimulus to trigger cell activation and/or cell death.

Interestingly, *A. actinomycetemcomitans* has the ability to invade vascular endothelial cells using platelet-activating factor as its receptor [47] although the bacterial adhesin mediating this interaction is not known. We recently reported that LtxA-mediated hemolysis can be blocked by gangliosides [48]. Gangliosides are glycoshingolipids on the surfaces of many cell types and are involved in signaling and membrane protein regulation [49]. We proposed that LtxA may have gained the ability to bind to gangliosides on red blood cells (RBC) because of the similarity of their sugar moieties and structure to the carbohydrate modification of LFA-1 [48]. Similar to our observations on endothelial cells, RBC lysis requires significantly higher doses of LtxA than needed for WBC killing [50]. Hence, it is possible that LtxA interacts with gangliosides on the surface of endothelial cells in a manner similar to that seen in RBCs.

In conclusion, we demonstrate that LtxA significantly increased expression levels of ICAM-1 and VCAM-1 in endothelial cells further corroborating pro-inflammatory effects of LtxA. Additionally, LtxA has important anti-proliferative as well as pro-apoptotic effects on microvascular endothelial cells and induces a G2/M phase cell cycle arrest. Apoptosis was partially caspase-dependent, however detailed mechanisms warrant further investigation. The presented work underlines not only the important function of LtxA in tissue destruction during *A. actinomycetemcomitans* infection but also its relevance in CVD.
